# Reversal of neutrophil-to-lymphocyte count ratio in early versus late death from septic shock

**DOI:** 10.1186/s13054-015-1144-x

**Published:** 2015-12-16

**Authors:** Florence Riché, Etienne Gayat, Romain Barthélémy, Matthieu Le Dorze, Joaquim Matéo, Didier Payen

**Affiliations:** Département d’Anesthésie - Réanimation - SMUR, Hôpitaux Universitaires Saint Louis – Lariboisière, Paris, France; UFR de Médecine, Université Paris Diderot, Paris, France; UMR-S 942, INSERM, Paris, France; UMR-S 1160, INSERM, Paris, France

## Abstract

**Introduction:**

Septic shock is one of the most frequent causes of admission to the intensive care unit (ICU) and is associated with a poor prognosis. Early and late death in septic shock should be distinguished because they may involve different underlying mechanisms. In various conditions, the neutrophil-to-lymphocyte count ratio (NLCR) has been described as an easily measurable parameter to express injury severity. In the present study, we investigated whether the timing of death was related to a particular NLCR.

**Methods:**

We conducted a prospective, single-center, observational study that included consecutive septic shock patients. Severity scores, early (before day 5) or late (on or after day 5 of septic shock onset) ICU mortality, and daily leukocyte counts were collected during the ICU stay. We assessed the association between leukocyte counts at admission and their evolution during the first 5 days with early or late death. The association between patient characteristics (including cell counts) and prognosis was estimated using Cox proportional cause-specific hazards models.

**Results:**

The study included 130 patients who were diagnosed with abdominal (*n* = 99) or extra-abdominal (*n* = 31) septic shock. The median (interquartile range) NLCR was 12.5 (6.5–21.2) in survivors and 6.2 (3.7–12.6) in nonsurvivors (*p* = 0.001). The NLCR at admission was significantly lower in patients who died before day 5 than in survivors (5 [3.5–11.6] versus 12.5 [6.5–21.2], respectively; *p* = 0.01). From day 1 to day 5, an increased NLCR related to an increase in neutrophil count and a decrease in lymphocyte count was associated with late death (+34.8 % [−8.2 to 305.4] versus −20 % [−57.4 to 45.9]; *p* = 0.003). Those results were present in patients with abdominal origin sepsis as well as in those with extra-abdominal sepsis, who were analyzed separately.

**Conclusions:**

In the present study, a reversed NLCR evolution was observed according to the timing of death. Septic shock patients at risk of early death had a low NLCR at admission, although late death was associated with an increased NLCR during the first 5 days.

**Electronic supplementary material:**

The online version of this article (doi:10.1186/s13054-015-1144-x) contains supplementary material, which is available to authorized users.

## Introduction

Although the rate has been reduced by compliance with the bundles recommended by the Surviving Sepsis Campaign, reported septic shock mortality still varies from 29 % to 38 % [1]. Both pro- and anti-inflammatory responses occur early and simultaneously in septic shock. A rapid and early upregulation of genes of the innate immune response occurs, along with downregulation of genes of the adaptive immune response [2–4]. This acute dysregulation may result in death in the first week (“early death”). This initial dysregulation may evolve to a complex state combined with an unabated innate immune response, which can lead to persistent, nonresolving inflammation, resulting in organ dysfunction and an impaired adaptive immune response that leaves the host unable to react to any assault [5–7]. This other type of dysregulation may lead to death within or beyond the first month (“late death”). In addition, several studies have suggested an even longer–term mortality due to sepsis with persistent inflammation associated with mortality over the course of 1 year [8–10]. The distinction between early (before day 5) and late (after day 5) death is increasingly being studied and may involve different underlying mechanisms [11–17]. Patients who die quickly must be taken into account in any study of sepsis and must not be excluded, because they represent a sizable ratio and are distinguished from those who may die after a longer period of time [18].

Polymorphonuclear neutrophils are the first cellular defense against infection and the key cell type of the innate immune system. The response to bacterial infection involves neutrophil recruitment and extravasation into the infected tissues. It can be seen as equilibrium between bone marrow release and tissue migration [19]. Lymphocytes are heterogeneous cell populations with different functional and phenotypical properties involved in adaptive immunity. Lymphopenia has been proposed as an indicator of mortality in severe sepsis, mainly because of its activation of apoptotic processes [20–22]. The physiological immune response of circulating leukocytes to various stressful events is often characterized by an increase in neutrophil counts and a decline in lymphocyte counts. Zahorec demonstrated a correlation between the severity of the clinical course and the grade of neutrophilia and lymphocytopenia [23]. He proposed the neutrophil-to-lymphocyte count ratio (NLCR), which is an easily measurable parameter to express injury severity. In the context of infections, the NLCR has been proven to predict bacteremia more accurately than routine parameters [24]. Using administrative data, researchers in a recent study suggested that the NLCR is associated with 28-day mortality in unselected intensive care unit (ICU) patients; however, they did not find this association when they focused on patients with sepsis [25]. Moreover, it seems that there is some association between the source of the infection and hospital mortality in patients with septic shock. Leligdowicz et al. [26] indicated that sepsis originating from the abdomen was associated with the highest rate of hospital mortality and that obstructive uropathy-associated urinary tract infections were associated with the lowest rate.

Accordingly, in the present study, we investigated whether the timing of death was related to a particular NLCR. In addition, we compared a group of patients with septic shock originating from the abdomen with a group who had sepsis of extra-abdominal origin.

## Methods

### Study design and ethical approval

We conducted a single-center prospective study in a surgical ICU. The protocol was approved by the institutional review board at our center (Comité d’Evaluation de l’Ethique des projets de Recherche Biomédicale Paris Nord, IRB 0006477).

### Patient selection

All consecutive patients with septic shock of abdominal or extra-abdominal origin admitted to the ICU from January 2011 to January 2014 were enrolled. All patients were admitted for septic shock, and data collection was started within 12 h of the onset of shock. Septic shock was defined according to the criteria of the American College of Chest Physicians/Society of Critical Care Medicine consensus conference [27]. Patients younger than 18 years of age, pregnant patients, and patients with aplasia or immunosuppressive disease (e.g., HIV) or receiving immunosuppressive therapy (chemotherapy, chronic used of steroids, autoimmune disease treatment) were excluded from the study. Of note, no patients received steroids during their ICU stay. All included patients were informed about the study and consented to participate. If the patient was unable to be informed, the next of kin was informed and provided consent for the patient to participate.

### Outcome measure

The main outcome measure was ICU mortality, which was categorized as early death (death from day 1 to day 4) or late death (death on or after day 5).

### Data collection

The following demographic and clinical data were collected: age, sex, comorbidities (Charlson comorbidity index score) [28], Simplified Acute Physiology Score II, Sequential Organ Failure Assessment score at day 1, length of ICU stay, ICU mortality, and associated secondary infections [29]. Circulating neutrophil and lymphocyte counts and the NLCR were determined at ICU admission and daily until discharge or death. When this count was performed several times per day, only the first measurement was considered. The normal ranges for the leukocyte counts in our central laboratory were 1.8–7 × 10^9^/L for neutrophils, 1–4 × 10^9^/L for lymphocytes, and NLCR = 2 (range, 0.45–7). Moreover, variations in cell counts and the NLCR from day 1 to day 5 were studied. Variations were defined as the relative difference in values between day 5 and day 1 (100 × [day 5 − day 1]/day 1).

### Statistical analysis

Continuous variables were expressed as the median (interquartile range), and categorical variables were expressed as the count (percentage). Patient characteristics were compared among several groups, namely survivors and early and late mortality, using the Kruskal-Wallis test (for continuous variables when more than two groups were compared), the Wilcoxon test (for continuous variables when two groups were compared), and the χ^2^ test (in all other cases). The main endpoint of the study was to investigate the association between the NLCR and ICU prognosis, with categories based on length of stay (from ICU admission to time of death or discharge). The cumulative incidence of ICU mortality over time was estimated, and alive at discharge was considering as a competing event. Associations between patient characteristics (including cell counts) and prognosis were estimated using Cox proportional cause-specific hazards models. Appropriate methods to analyze the censored data were used. The ability of cell counts to discriminate survivors from nonsurvivors was studied using receiver operating characteristic (ROC) curves and areas under the ROC curve (AUC). Statistical analyses were performed using R statistical software (http://www.r-project.org/). A two-sided *p* value <0.05 was considered statistically significant.

## Results

**Fig. 1:**
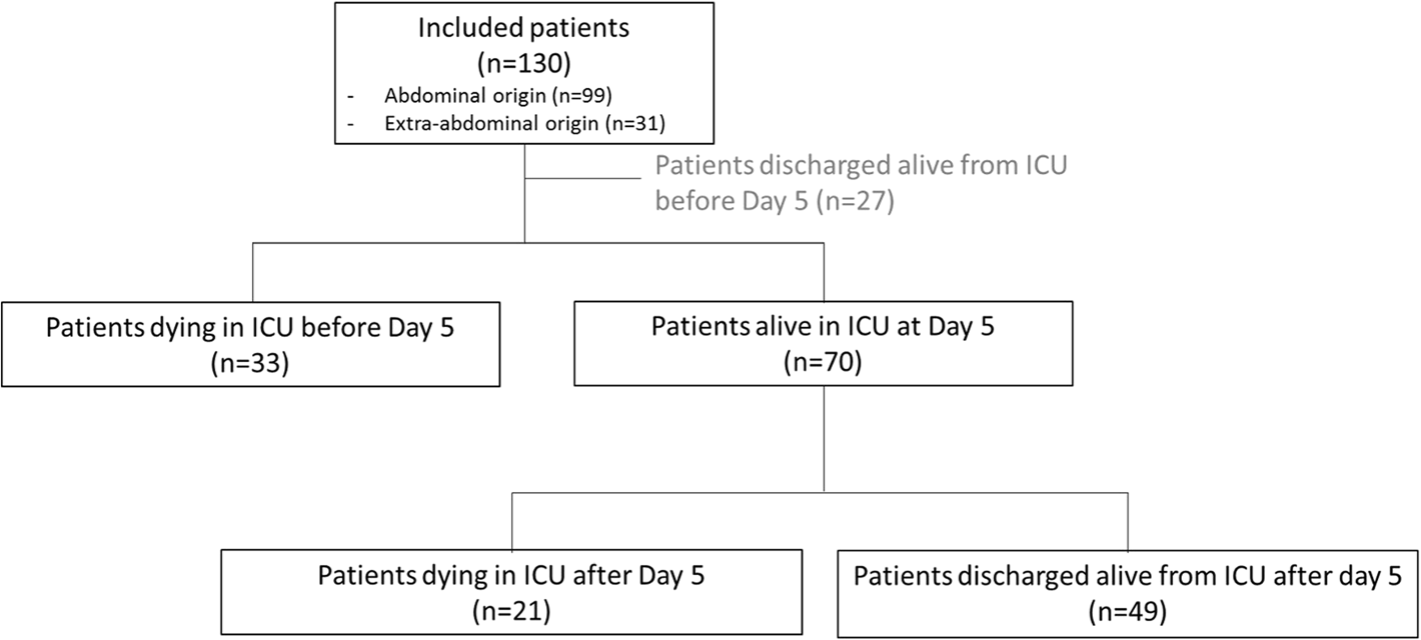
Flowchart depicting the study process. *ICU* intensive care unit

Table 1 shows the baseline characteristics of the cohort of 130 septic shock patients. Septic shock that originated in the abdomen was present in 99 patients (75 %). Among the 130 patients, 117 (90 %) benefited from bacteriological sampling from the nidus of infection and 22 (17 %) of those presented with bacteremia at admission. Of the sample, 22 % were sterile, and multiple microorganisms were found in 8 %. Gram-negative bacilli were identified in 47 % of the cases, gram-positive cocci in 34 %, and yeast in 8 %. Overall, ICU mortality was 41 % (54 of 130 patients), with 61 % (33 of 54) of the deaths occurring during the first 4 days (the “early-death group”) and 39 % (21 of 54) occurring on or after day 5 (the “late-death group”) (Fig. 1).

**Table 1:** Patient characteristics

	All patients (*n* = 130)	Abdominal septic shock (*n* = 99) (1)	Extra-abdominal septic shock (*n* = 31) (2)	*p* Value (1) vs (2)
Age, yr	72.7 (60.3–82.4)	72.5 (61.4–82)	74 (58.7–84.2)	0.76
Female	54 (41.5)	42 (42.4)	12 (38.7)	0.54
Male	76 (58.5)	57 (57.6)	19 (61.3)	0.54
Origin of sepsis				
Digestive tract	99 (74.6)	99 (100)	0 (0)	
Bone and soft tissue	14 (11)	0 (0)	14 (45)	
Pulmonary	7 (5)	0 (0)	7 (22)	
Urinary tract	6 (4)	0 (0)	6 (19)	
Blood/endocarditis	4 (3)	0 (0)	4 (12)	
Comorbidities				
CAD	24 (18)	20 (20)	4 (13)	0.36
Diabetes	28 (21)	23 (23)	5 (16)	0.4
COPD	13 (10)	8 (8)	5 (16)	0.19
Cancer/immunosuppression	33 (25)	28 (28)	5 (16)	0.17
Charlson comorbidity index score	5 (4–6)	5 (4–6)	5 (4–6)	0.86
Severity and outcome				
SAPS II	52.5 (42–60.8)	52 (40.5–61)	53 (44–59)	0.61
SOFA score at admission	8 (6–12)	8 (6–11.5)	9 (8–12)	0.066
In-ICU LOS	5 (2–11.8)	5 (2–10)	10 (3–18)	0.061
In-ICU mortality	54 (41.5)	37 (37.4)	17 (54.8)	0.085
Hospital-acquired infection	29 (22.3)	20 (20.2)	9 (29)	0.3
Cell counts at admission				
Neutrophils, 10^9^/L	9.7 (4–16.3)	10.1 (3.9–16.3)	8.3 (5.8–17.1)	0.78
Lymphocytes, 10^9^/L	0.8 (0.5–1.3)	0.8 (0.5–1.3)	0.9 (0.6–1.4)	0.88
NLCR	9.3 (4.8–18.2)	9 (4.6–17.9)	11.5 (5.5–18.5)	0.47

*Abbreviations*: *CAD* coronary artery disease, *COPD* chronic obstructive pulmonary disease, *SAPS II* Simplified Acute Physiology Score II, *SOFA* Sequential Organ Failure Assessment, *ICU* intensive care unit, *LOS* length of stay, *NLCR* neutrophil-to-lymphocyte count ratio

Continuous variables are expressed as median with interquartile range or count with percentage, as appropriate

### Admission circulating neutrophil and lymphocyte counts and NLCRs of survivors versus nonsurvivors

Considering the cohort of 130 patients, the neutrophil count at admission was similar between survivors and nonsurvivors. The lymphocyte count was higher (*p* = 0.002) with a reduced NLCR (*p* = 0.0014) in the nonsurvivor group (Table 2). Differentiation of early and late death revealed the NLCR at admission of early-death patients was significantly lower than that of both late-death patients and survivors (*p* = 0.01) (Table 3). Of note, the NLCR was higher in patients with bacteremia at admission (13.9 [9.1 to 31.2] versus 8.8 [4.6 to 16.4]; *p* = 0.024).

**Table 2:** Patient characteristics according to survivors and nonsurvivors

	All patients		
	Survivors (*n* = 76)	Nonsurvivors (*n* = 54)	*p* Value
Age, yr	70.7 (57.9–81.7)	75.7 (64.6–83.3)	0.095
Female	31 (40.8)	23 (42.6)	0.84
Male	45 (59.2)	31 (57.4)	
Comorbidities			
CAD	14 (18.4)	10 (18.5)	0.99
Diabetes	16 (21.1)	12 (22.2)	0.87
COPD	8 (10.5)	5 (9.3)	0.81
Cancer/immunosuppression	19 (25)	14 (25.9)	0.9
Charlson comorbidity index score	5 (3–6)	5 (4–6.8)	0.052
Severity and outcome			
SAPS II	44 (39.8–54)	59.5 (53–70)	<0.0001
SOFA score at admission	7 (5.8–9)	12 (7–14.8)	<0.0001
In-ICU LOS	7 (4–11.2)	3 (1.2–11.8)	0.0049
Hospital-acquired infection	12 (15.8)	17 (31.5)	0.034
Cell counts at admission			
Neutrophils, 10^9^/L	9.7 (5.5–16.5)	9.8 (3.2–16.2)	0.43
Lymphocytes, 10^9^/L	0.7 (0.5–1.1)	1.1 (0.7–1.9)	0.0022
NLCR	12.5 (6.5–21.2)	6.2 (3.7–12.6)	0.0014

*Abbreviations*: *CAD* coronary artery disease, *COPD* chronic obstructive pulmonary disease, *SAPS II* Simplified Acute Physiology Score II, *SOFA* Sequential Organ Failure Assessment, *ICU* intensive care unit, *LOS* length of stay, *NLCR* neutrophil-to-lymphocyte count ratio

Continuous variables are expressed as median with interquartile range or count with percentage, as appropriate

**Table 3:** Patient characteristics according to survivors, early death, and late death

	Survivors (*n* = 76) (1)	Early death (*n* = 33) (2)	Late death (*n* = 21) (3)	*p* Value (2) vs (1 + 3)	*p* Value (2) vs (3)
Age, yr	70.7 (57.9–81.7)	74.7 (65–81.9)	77.9 (64.4–85.7)	0.57	0.38
Female	31 (40.8)	14 (42.4)	9 (42.9)	0.9	0.97
Male	45 (59.2)	19 (57.6)	12 (57.1)		0.97
Comorbidities					
CAD	14 (18.4)	7 (21.2)	3 (14.3)	0.64	0.51
Diabetes	16 (21.1)	5 (15.2)	7 (33.3)	0.30	0.12
COPD	8 (10.5)	3 (9.1)	2 (9.5)	0.84	0.96
Cancer/immunosuppression	19 (25)	8 (24.2)	6 (28.6)	0.86	0.73
Charlson comorbidity index score	5 (3–6)	5 (4–6)	5 (4–7)	0.15	0.97
Severity and outcome					
SAPS II	44 (39.8–54)	64 (56–76)	55 (49–61)	<0.0001	0.003
SOFA score at admission	7 (5.8–9)	13 (10–16)	8 (6–9)	<0.0001	0.0004
In-ICU LOS	7 (4–11.2)	2 (1–2)	14 (10–22)	<0.0001	<0.0001
Hospital-acquired infection	12 (15.8)	3 (9.1)	14 (66.7)	0.035	<0.0001
Cell counts at admission					
Neutrophils, 10^9^/L	9.7 (5.5–16.5)	6.9 (2.5–16.3)	10.9 (6.1–13.2)	0.15	0.25
Lymphocytes, 10^9^/L	0.7 (0.5–1.1)	0.9 (0.6–1.6)	1.2 (0.9–2)	0.35	0.17
NLCR	12.5 (6.5–21.2)	5 (3.5–11.6)	7 (4–12.9)	0.011	0.41

*Abbreviations*: *CAD* coronary artery disease, *COPD* chronic obstructive pulmonary disease, *SAPS II* Simplified Acute Physiology Score II, *SOFA* Sequential Organ Failure Assessment, *ICU* intensive care unit, *LOS* length of stay, *NLCR* neutrophil-to-lymphocyte count ratio

Continuous variables are expressed as median with interquartile range or count with percentage, as appropriate

### Circulating neutrophils, lymphocytes, and NLCR variations from admission to day 5 in survivor and late-death groups

**Fig. 2:**
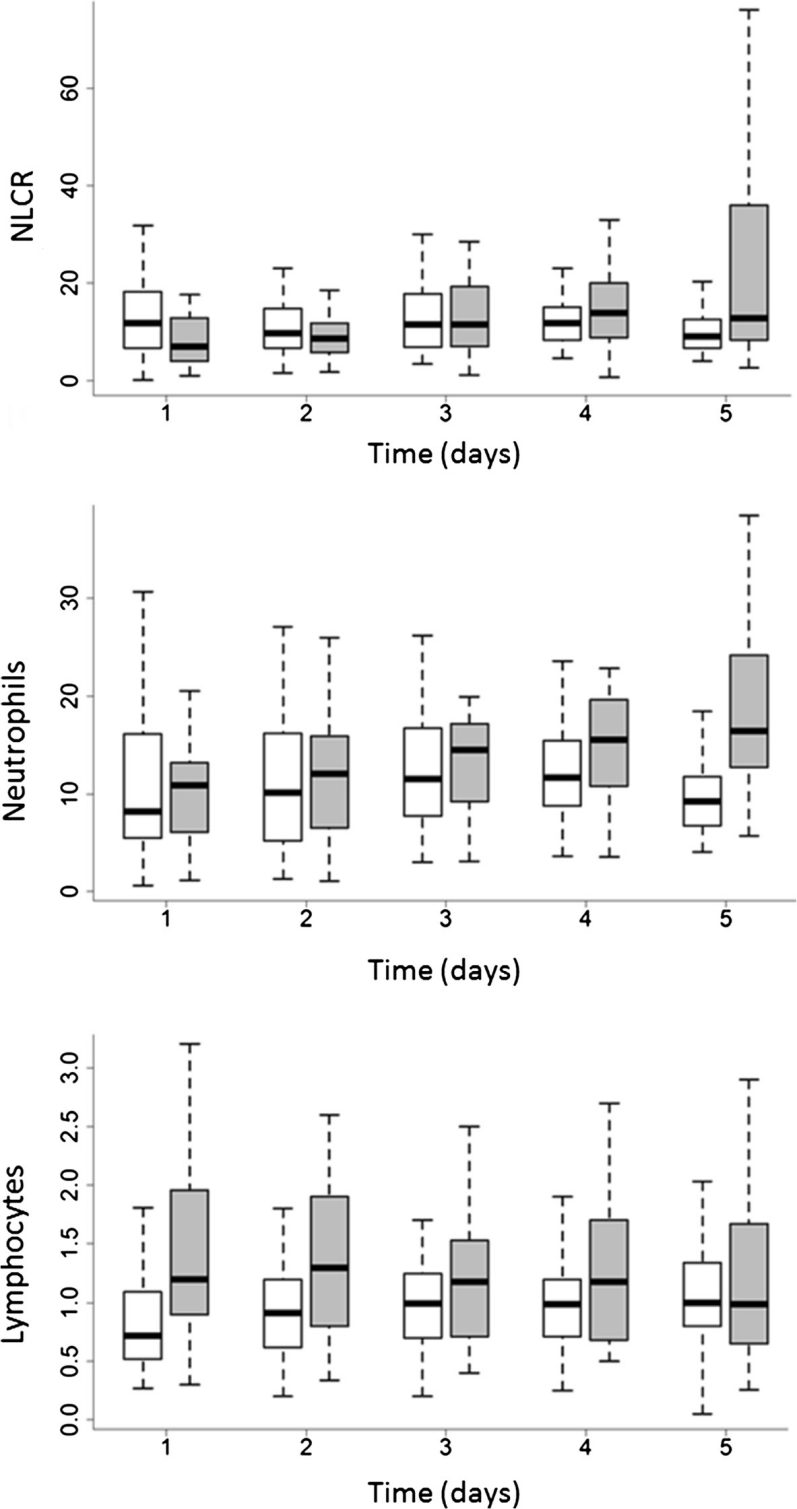
Daily values of circulating neutrophils, lymphocytes, and neutrophil-to-lymphocyte count ratio (NLCR) from day 1 to day 5. *Shaded bars* represent nonsurvivors; *open bars* indicate survivors. *p* Values derived from two-way analysis of variance are reported in Additional file 1: Table S1. The box depict median value (thick bar) with 25the 75th, 10th and 90th percentiles

Among the patients alive at day 5, patients who died later (late-death group) were compared with survivors. At day 5, 70 patients were alive, and 21 of those subsequently died. In the late-death group, the variations in the circulating neutrophil and lymphocyte counts from day 1 to day 5 were compared with those of survivors. The neutrophil increase (difference between day 5 and day 1) was significant (*p* = 0.026), with a decreased lymphocyte count (*p* = 0.034) and a subsequent increase in the NLCR (*p* = 0.003) associated with the risk of late death (Table 4). The daily values for cell counts from day 1 to day 5 are shown in Fig. 2 and Additional file 1: Table S1.

**Table 4:** Neutrophil, lymphocyte, and NLCR variations from admission to day 5 in late survivors and late nonsurvivors

	Survivors after day 5 (*n* = 49)	Late death after day 5 (*n* = 21)	*p* Value
Age, yr	72.5 (61.6–79.3)	77.9 (64.4–85.7)	0.2
Female	16 (32.7)	9 (42.9)	0.41
Male	33 (67.3)	12 (57.1)	
Comorbidities			
CAD	12 (24.5)	3 (14.3)	0.34
Diabetes	11 (22.4)	7 (33.3)	0.34
COPD	7 (14.3)	2 (9.5)	0.59
Cancer/immunosuppression	10 (20.4)	6 (28.6)	0.46
Charlson comorbidity index score	5 (3–6)	5 (4–7)	0.66
Severity and outcome			
SAPS II	48 (42–56)	55 (49–61)	0.083
SOFA score at admission	9 (6–10)	8 (6–9)	0.58
In-ICU LOS	10 (7–18)	14 (10–22)	0.15
Hospital-acquired infection	12 (24.5)	14 (66.7)	0.00082
Cell counts at admission			
Neutrophils, 10^9^/L	8.2 (5.5–16.1)	10.9 (6.1–13.2)	0.68
Lymphocytes, 10^9^/L	0.7 (0.5–1.1)	1.2 (0.9–2)	0.0012
NLCR	11.8 (6.6–18.3)	7 (4–12.9)	0.06
Cell counts at day 5			
Neutrophils, 10^9^/L	9.2 (6.7–11.8)	16.4 (13.3–24.2)	0.0001
Lymphocytes, 10^9^/L	1 (0.8–1.3)	1 (0.7–1.3)	0.89
NLCR	9.1 (6.7–12.5)	12.8 (8.4–35.4)	0.028
Cell counts variations from day 1 to day 5			
Neutrophils, %	6.3 (−47.2 to 75.3)	50.5 (3.4 to 207.0)	0.026
Lymphocytes, %	33.3 (−20.0 to 141.7)	−21.8 (−43.2 to 44.6)	0.034
NLCR, %	−20.0 (−57.4 to 45.9)	34.8 (−8.2 to 305.4)	0.003

*Abbreviations*: *CAD* coronary artery disease, *COPD* chronic obstructive pulmonary disease, *SAPS II* Simplified Acute Physiology Score II, *SOFA* Sequential Organ Failure Assessment, *ICU* intensive care unit, *LOS* length of stay, *NLCR* neutrophil-to-lymphocyte count ratio

### Discriminatory threshold of early and late death

**Fig. 3:**
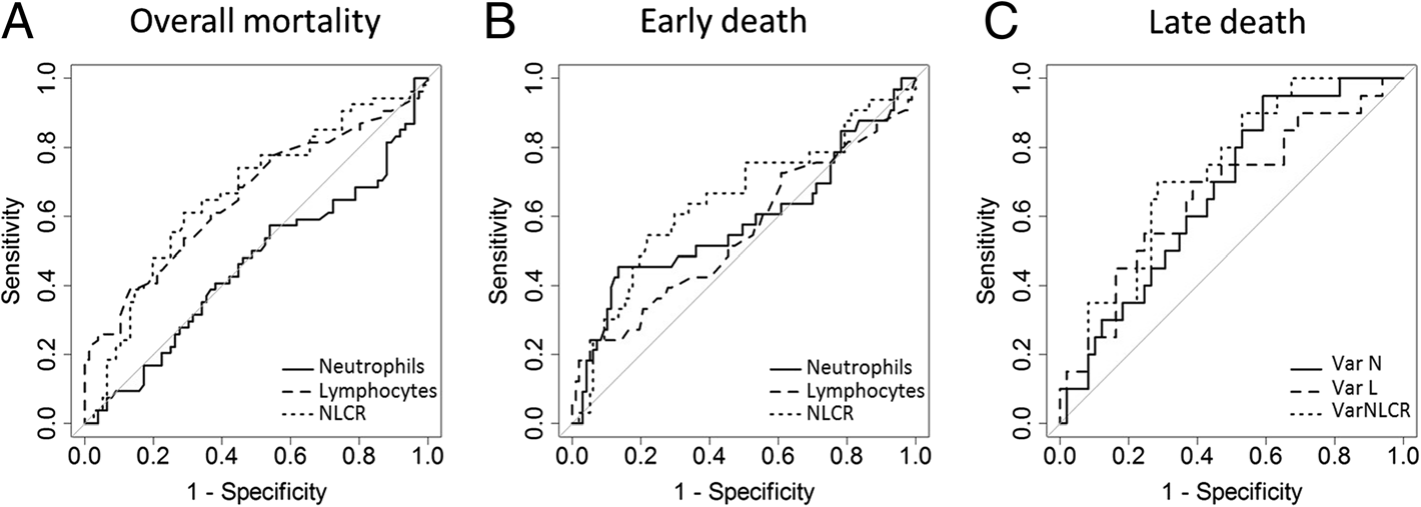
Receiver operating characteristic curves for the best neutrophil-to-lymphocyte count ratio (NLCR) thresholds to predict overall mortality (**a**), early death (**b**), and late death (**c**). Areas under the curve with 95 % confidence intervals are displayed in Additional file 2: Table S2

The discriminatory ability of neutrophils, lymphocytes, and the NLCR to predict early or late death is shown in Fig. 3. The AUCs for NLCRs were 0.7, 0.65, and 0.72 for overall, early death, and late death, respectively. The most accurate discriminatory NLCR thresholds were 7.7 (sensitivity = 0.71, specificity = 0.61) for overall mortality, 5.3 (sensitivity = 0.78, specificity = 0.55) for early death, and an increase of 18 % in the NLCR for late death (see Additional file 2: Table S2).

Of note, these results were present both in patients with sepsis of abdominal origin and in those with extra-abdominal sepsis, who were analyzed separately (see Additional file 3: Table S3 and Additional file 4: Figures S4 and S5).

## Discussion

In the present study, we found that at the onset of septic shock, a low NLCR was associated with nonsurvival. This low NLCR was due to a higher lymphocyte count. When distinguishing time until death in the early-death (before day 5) and late-death (on or after day 5) groups, the NLCR of the early-death patients at admission was significantly lower than that of late-death patients and survivors. The risk of late death was associated with a neutrophil increase, lymphocyte count decrease, and subsequent increase in the NLCR from day 1 to day 5.

Early death (before day 5) versus late death (on or after day 5) is increasingly being studied [5, 6, 11, 14–17]. An overly strong immune response with a cytokine storm [2], mitochondriopathy, and massive tissue damage may lead to early death [3]. This dysregulation may cause a complex state combining an unabated innate immune response that leads to persistent inflammation, resulting organ dysfunction, and an impaired adaptive immune response that leaves the host unable to react to any assault [2, 6]. In our study, clinical characteristics did not differ between early- and late-death patients, particularly age, severity scores, and comorbidities. The most remarkable clinical difference was that of hospital-acquired infections, which was 2.7 times higher in nonsurvivors. Although nonsignificant, the ICU length of stay was longer in nonsurvivors. Whether the hospital-acquired infection was a consequence of alteration of adaptive function related to lymphopenia, whether it was responsible for neutrophilia, or both remains unclear.

Most of the prognostic scores mention leukocytosis (>12,000/mm^3^) or leukopenia (<4000/mm^3^) as a severity index, but none considers the leukocyte subpopulations [30–34]. Significant differences between neutrophil and lymphocyte counts and, consequently, their ratio have been shown to predict the severity or outcome in different pathological circumstances, such as surgical stress, systemic inflammation and sepsis [23], bacteremia [24], community-acquired pneumonia [35], ischemic events [36], and cancer [37, 38]. We must be cautious because the prognostic value of the NLCR in our study is below the value of 0.8 that is felt to be indicative of a reliable prognostic marker.

The evolution of these leukocyte populations may differ based on their respective role in the inflammatory response. Neutrophils are the first cellular line of defense in the innate immune system and have a short half-life, while lymphocytes are the major cells of the adaptive immune system.

In our study, all patients were lymphopenic. Lymphopenia is due first to a huge recruitment from peripheral circulation to the nidus of infection, followed by lymphocyte apoptosis caused by several stimuli [20]; persistent lymphopenia of the late-death patients is most likely due to ongoing sepsis-induced lymphocyte apoptosis secondary to the continued release of proapoptotic stimuli and the nonresolution of inflammation.

Among the patients with lymphopenia, the lymphocyte count was higher in all nonsurvivors than in survivors. This difference was not found when all nonsurvivors were separated into early- and late-death groups, probably because of the small number of patients in each group. Nevertheless, the NLCR was surprisingly reduced in patients who died early, independent of the origin of the sepsis. This result might be explained by a decrease in neutrophils associated with an increased lymphocyte count. Some researchers have not reported this result, likely because they included only patients who survived until day 4 hospital mortality without considering death in the first 5 days [18]. Accounting for death in the first 5 days is a significant issue. In the present study, more than two-thirds of the patients died in the first 5 days, which is a high rate and cannot be ignored. Although lymphocyte apoptosis is well described in septic shock, some authors have demonstrated that the early phase of human sepsis is characterized by a combination of apoptosis and the proliferation of T cells. A rapid recovery of total, CD4^+^, and CD8^+^ T lymphocytes could indicate their intense trafficking between tissues and the lymphatic system during the acute phase of illness [12, 39–42]. Moreover, patients who die early might produce higher levels of stress hormones such as adrenaline, which increases lymphocyte counts [43]. Although the exact underlying mechanism for a higher lymphocyte count in nonsurvivors remains unclear and requires further investigation, the NLCR appears to be a simple parameter to detect patients at risk of early death.

An increase in the NLCR from day 1 to day 5, with an increase in the neutrophil count and a smooth drop in the lymphocyte count, was associated with late death in our study population. Interestingly, in other studies researchers have found that late mortality, at 6 months or 12 months after emergency abdominal surgery, was associated with a persistent high neutrophil count [44]. Increased neutrophils might indicate that the site of the infection has not been eradicated and that there is still pus or abscess in the peritoneal cavity or other nidus. Thus, the bone marrow continues to produce large amounts of neutrophils to fight the infection. We also suggest that the neutrophils might not become apoptotic. In contrast to lymphocytes, neutrophil apoptosis is beneficial in sepsis. The apoptosis of these cells initiates and facilitates the resolution of inflammation, tissue repair, and reestablishment of homeostasis. Apoptotic neutrophils are cleared by macrophages and prompt the macrophages to switch from a proinflammatory to an anti-inflammatory phenotype [21, 45, 46]. This neutrophilic pattern could reflect a nonresolving inflammation [47].

Finally, while some studies have indicated that the source of infection is important to consider because the immune response differs according to the site [26], we did not find any significant differences between the abdominal and extra-abdominal sepsis groups concerning lymphocytes, neutrophil counts, or the NLCR in the early- or late-death groups. However, the size of the extra-abdominal sepsis group was quite small compared with the abdominal sepsis group.

### Limitations of the study

The present study has several limitations. First, we conducted a single-center observational study, and thus, as with any observational study, the potential remains for residual confounding. The results have to be confirmed in other centers. Second, for some patients, several measures were available on the same day, and we always used the first one to maximize to consistency among the patients. However, we could have missed information related to intraday cell count variations. Third, we analyzed circulating neutrophil and lymphocyte counts and did not explore the different subsets of lymphocytes. Phenotypic markers are missing, but this is the subject of a forthcoming study. Fourth, our sample size was limited, and our results should be confirmed in a larger population. Given our limited sample size, we were not able to study other time points to define late death, such as death after 14 days or 28 days. Finally, no information about inflammatory biomarkers (such as C-reactive protein or procalcitonin) was available in our patients; the role of these markers in this context and their potential interaction with NLCR should be assessed.

## Conclusions

This study demonstrates a clear relationship between the NLCR and the risk of death in septic shock patients. A reversed NLCR evolution was observed according to the timing of death. Septic shock patients at risk of early death presented a low NLCR at admission, although late death was associated with an increased NLCR during the first 5 days. Early and late death should be distinguished because they may involve different underlying mechanisms, and the NLCR might be considered as a discriminant indicator of early or late death.

In addition, our findings provide more insight into biology. The circulating neutrophil and lymphocyte trends observed in this study offer an interesting mechanistic viewpoint. We observed that circulating lymphocytes and the NLCR behave in opposite ways in early- and late-death patients, supporting the hypothesis that divergent mechanisms could be involved in these two groups.

## Key messages

- In patients admitted to the ICU for septic shock, a low NLCR at admission is associated with a risk of early death.
- In the same population, an increase in the NLCR during the first 5 days is associated with a risk of late death.
- Early and late death should be distinguished because they may involve different underlying mechanisms.

## Additional files

Additional file 1: Table S1.
*p* Values of the two-way ANOVA comparing cellular counts over time between survivors and nonsurvivors. (DOC 21 kb) Additional file 2: Table S2. Area under the receiver operating characteristic (ROC) curve for cellular counts to predict death. (DOC 21 kb) Additional file 3:
**Table S3.** Neutrophils, lymphocytes counts and NLCR variations according to the septic origin ( abdominal vs extra-abdominal) and death timing. (DOC 21 kb) Additional file 4:
**Figure S4.** In-ICU early death cumulative incidence of abdominal (n=99) and extra-abdominal (n=31) septic shock groups **Figure S5**. Cumulative incidence of death for patients alive at day 5 and still hospitalized in ICU according to the variation from Day 1-to-5 of NLCR. (DOC 21 kb)

## Abbreviations

AUC: area under the receiver operating characteristic curve
ICU: intensive care unit
NLCR: neutrophil-to-lymphocyte count ratio
ROC: receiver operating characteristic
SAPS II: Simplified Acute Physiology Score II
SOFA: Sequential Organ Failure Assessment
